# Cell-cell contact and specific cytokines inhibit apoptosis of colonic epithelial cells: growth factors protect against c-myc-independent apoptosis.

**DOI:** 10.1038/bjc.1997.167

**Published:** 1997

**Authors:** A. Hague, D. J. Hicks, T. S. Bracey, C. Paraskeva

**Affiliations:** CRC Colorectal Tumour Biology Research Group, Department of Pathology and Microbiology, School of Medical Sciences, Bristol, UK.

## Abstract

**Images:**


British Journal of Cancer (1997) 75(7), 960–968
© 1997 Cancer Research Campaign

### Cell–cell contact and specific cytokines inhibit apoptosis of colonic epithelial cells: growth factors protect against c-*myc*-independent apoptosis

**A Hague, DJ Hicks, TS Bracey and C Paraskeva**

CRC Colorectal Tumour Biology Research Group, Department of Pathology and Microbiology, School of Medical Sciences, University Walk, Bristol BS8 1TD, UK

**Summary** In this study we sought factors that determine the survival of human colonic epithelial cells. Normal colonic epithelial cells are dependent on cell–cell contacts and survival factors for the inhibition of apoptosis whereas, during colorectal tumorigenesis, cells develop mechanisms to evade these controls. The ability to survive loss of cell–cell contacts and/or growth factor deprivation is a marker of tumour progression. Many adenoma (premaligant) cultures survive only if cell–cell contacts are maintained in vitro and die by apoptosis if trypsinized to single cells. This also occurs in adenomas derived from familial adenomatous polyposis (FAP) patients, therefore APC mutations do not confer resistance to cell death in response to loss of cell–cell contacts. We show here that if cell–cell contacts are maintained such cells are capable of survival in suspension. Adenoma cells also undergo apoptosis in response to removal of serum and growth factors from the medium. After removal of serum and growth factors c-*myc* is down-regulated within 2 h. Therefore, the induction of apoptosis is not an inappropriate response of the cells due to a deregulated c-*myc* gene. The apoptotic response is also p53 independent. Such cultures have been used to determine specific survival factors for colonic epithelial cells. Insulin, the insulin-like growth factors I and II, hydrocortisone and epidermal growth factor (EGF) protect cells from the induction of apoptosis in the absence of serum over a short-term period of 24 h. This approach may give insight into the factors governing growth and survival of colonic epithelial cells in vivo. This is the first report of specific growth factors protecting against apoptosis in human colonic epithelial cells.

**Keywords:** apoptosis; colon; survival factors; insulin-like growth factors; c-*myc*

It has been proposed that most mammalian cells are programmed to undergo apoptosis unless they are continuously signalled by other cells not to do so (Raff, 1992). Cell death by apoptosis is thus considered as a default pathway, and withdrawal of specific factors that physiologically support the survival of the cell type can induce characteristic changes associated with apoptosis, including cell shrinkage, chromatin condensation and internucleosomal fragmentation (Wyllie, 1980). Apoptosis can be induced in hormone-dependent tumour cells; for example, prostate carcinoma cells apoptose after androgen depletion (Kyprianou et al, 1990) and breast carcinoma cells apoptose in response to oestrogen depletion (Kyprianou et al, 1991). In addition, various cytokines may regulate cell survival and the survival factors required are specific to the tissue type. For example, colony-stimulating factor is a survival factor for haematopoietic precursor cells (Williams et al, 1990), granulocyte–macrophage colony-stimulating factor for human polymorphonuclear neutrophils (Brach et al, 1992), nerve growth factor (NGF) for neural cells (Mesner et al, 1992), erythropoietin, stem cell factor and insulin-like growth factor I (IGF-I) for erythroid progenitor cells (Muta and Kranz, 1993) and fibroblast growth factor for vascular endothelial cells (Araki et al, 1990). In all of these cases, the protection conferred is by inhibition of apoptosis. Barres et al (1992) assessed the ability of various cytokines to act as survival factors for oligodendrocytes and their precursors isolated from the rat optic nerve. For the progenitor cells, both insulin-like growth factors, IGF-I and-II, and platelet-derived growth factor (PDGF) acted as survival factors, whereas mature oligodendrocytes have lost their PDGF receptors and can no longer be rescued by PDGF, but are still rescued by the IGFs. These observations suggest not only that the survival factors are specific to the tissue type, but also that the survival requirements of a cell may change during maturation.

Received 26 July 1996
Revised 25 September 1996
Accepted 2 October 1996

Correspondence to: A Hague

Although there have been reports of specific survival factors for a number of other tissues, surprisingly little work has been done in the colon, and there have been no previous reports of specific cytokines acting as survival factors in colonic epithelial cells. As the maintenance of cell number is critical for normal tissue homeostasis and a breakdown in the control of cell numbers could be a critical event during carcinogenesis, the identification of specific survival factors could be important for our understanding of the process of colorectal carcinogenesis.

In the colonic epithelium, there must be strict controls on cell survival in the maintenance of crypt length and architecture. In the colonic crypt, cell proliferation in the lower two-thirds of the crypt is balanced by an equal rate of cell loss at the luminal surface. This loss may be due to a passive detachment of the cells into the colonic lumen or may be occurring by an active process of programmed cell death or apoptosis. Evidence for the involvement of apoptosis in the loss of cells at the luminal surface has been provided by Hall et al (1994) by scoring morphologically apoptotic cells and relating them to their position in the colonic crypt. Hall et al (1994) demonstrated that most of the apoptotic bodies occurred in the non-proliferative compartment. Calculations of the number of apoptoses per crypt per day provided evidence that apoptosis can effectively offset the rate of new cell production. Gavrieli et al (1992) and Bedi et al (1995) reported DNA breaks in the cells towards the top of the crypt by the use of in situ end-labelling techniques.

Epithelial cells from normal adult human colon are notoriously difficult to culture. Buset et al (1987) have reported the short-term culture (2–4 days) of normal colonic epithelial cells, and our experience with such primary cultures is similar (Paraskeva and Williams, 1992). Isolated colonocytes only survive for a few days in culture. In addition, there are few reports of successful establishment of premalignant colorectal adenoma cell lines; however, the serial passage of adenoma-derived cells is possible if cell–cell contacts are maintained (Paraskeva et al, 1984; Willson et al, 1987; Whitehead et al, 1991). Adenoma cells can be routinely passaged using disease, an enzyme which removes cells as clumps rather than single cells and retains cell–cell contacts. This permits growth of cells from smaller adenomas that are lost if passaged with trypsin. We have previously shown that an important marker of tumour progression in colorectal carcinogenesis is the ability of cells to grow after single-cell trypsinization (clonogenicity), i.e. normal colonocytes and early adenomas cannot be passaged using trypsin, whereas carcinomas and some later stage adenomas can (Paraskeva et al, 1989*a* Williams et al, 1990). It has been shown by Bates et al (1994) that colonic epithelial cells undergo apoptosis in response to inhibition of intercellular contact by anti-integrin antibodies. We show here that our non-clonogenic adenoma cell lines die by apoptosis if subjected to trypsinization.

In addition to survival factors mediated by cell–cell contact, the serum added to the medium to enable proliferation of the cells also provides factors for survival. On withdrawal of the serum and growth factors from the medium, there is an increase in the extent of apoptosis in some colorectal tumour cell cultures. This system was used to establish if specific cytokines were able to act as survival factors for colonic epithelial cells, rescuing the cells from apoptosis induced by serum withdrawal.

### METHODS

### Standard culture conditions

The cell lines were cultured as described in Paraskeva et al (1984). Cells were grown in Dulbecco's modified Eagle medium (DMEM) supplemented with 20% fetal bovine serum (FBS) (batch selected), 2 mM glutamine, 0.2 units $ml^{-1}$ insulin (human Actrapid insulin (0.2 IU $ml^{-1}$=7.692 μg $ml^{-1}$) Novo Nordisk Pharmaceuticals, Chartres, France), 1 μg $ml^{-1}$ hydrocortisone sodium succinate, 100 units $ml^{-1}$ penicillin and 100 μg $ml^{-1}$ streptomycin.

### Cell lines

PC/AA, PC/BH and S/AN were used as examples of non-clonogenic adenoma cell lines that do not grow after trypsinization (Paraskeva et al, 1984, 1989*a*). These cells are routinely grown on type IV collagen-coated flasks and are passaged as clumps of cells using the neutral protease disease. These cells were used to determine the fate of the cells after trypsinization and to examine the importance of cell–cell contacts for culture survival.

The adenoma cell lines AA/C1 and RG/C2 are clonogenic variants of the parental cell lines PC/AA and S/RG, respectively, and are non-tumorigenic. They are routinely grown on plastic and are passaged using trypsin. AA/C1 was a rare variant of the cell line PC/AA (Williams et al, 1990) and therefore probably represents a later stage in tumour progression. In contrast, the S/RG cell line grew readily after initial trypsinization (Paraskeva et al, 1989*a*), in vitro evidence that it may have been derived from a more advanced adenoma. The two clonogenic adenoma cell lines and a carcinoma cell line PC/JW/FI (Berry and Paraskeva, 1988) were used to determine whether the cells require serum for survival in culture.

### Induction of apoptosis by disruption of cell–cell contacts and cell–matrix interactions

The cell lines PC/AA and S/AN were routinely passaged using dispase, which removes the cells as clumps or sheets and retains cell–cell contacts. In a series of experiments, these cell lines were subjected to trypsinization to determine the fate of the cells. The cells were prevented from readhering by plating onto bacteriological Petri dishes coated with polyHEMA (Frisch and Francis, 1994) at a seeding density of $5 \times 10^5$ viable (trypan blue negative) cells per 6-cm dish. Samples were taken at various times after trypsinization and the cells were examined for apoptotic morphology by fluorescence microscopy of acridine orange-stained cells. As, in these experiments, apoptosis could be resulting from disruption of cell–cell contacts or from depriving the cells of a suitable substrate, trypsinized PC/AA cells (passage 19) were also seeded on collagen-coated Petri dishes. Parallel flasks of PC/AA were removed as clumps of cells using dispase and the same number of cells seeded onto either polyHEMA or collagen-coated Petri dishes.

### Apoptosis after serum depletion

Three clonogenic cell lines were used for these studies: the two adenoma cell lines AA/C1 (a clonogenic derivative of PC/AA) and RG/C2 (a clonogenic derivative of S/RG) and a carcinoma cell line PC/JW/FI. Cells were seeded at a density of $10^6$ cells per T25 flask. Three days after seeding, once the cells were in exponential growth, the medium was replaced. The test cells were fed with standard medium containing 0.5% fetal bovine serum. At varying times after serum depletion (48, 72 and 96 h), the cultures were assessed for the extent of apoptosis. As shown previously (Hague et al, 1993; Wagner et al, 1993; Bracey et al, 1995), the proportion of non-adherent or floating cells in the flasks can be used as a measure of the extent of apoptosis, in conjunction with examination of the cells by acridine orange staining to demonstrate that the majority of the floating cell population consists of apoptotic cells. The floating cells were collected and the adherent cells removed by trypsinization. The numbers of floating and attached cells per flask were counted separately and then each sample was stained with acridine orange to determine the proportion of cells that were apoptotic. Unfixed cells were stained with acridine orange 5 μg $ml^{-1}$ in phosphate-buffered saline (PBS) for 10 min before examination by fluorescence microscopy.

### Acridine orange–propidium iodide dual staining

For assessment of apoptosis after trypsinization of the PC/AA adenoma cell line, acridine orange–propidium iodide dual staining of unfixed cells was used. The mixture of fluorescent dyes consisted

© Cancer Research Campaign 1997

of acridine orange at 5 μg ml$^{-1}$ and propidum iodide at 5 μg ml$^{-1}$ in PBS. These dyes stain the DNA and allow visualization of the condensed chromatin of apoptotic cells. Slides were observed under a fluorescence microscope with a HBO 100 lamp. Propidium iodide was visualized by standard rhodamine excitation (excitation wavelengths between 515 and 560 nm and barrier filter LP590). Acridine orange was observed using standard narrow-band FITC excitation (excitation wavelengths 450–490 nm and barrier filter 520–560 nm). Propidium iodide only stains cells in late stages of apoptosis and secondary necrosis when membrane integrity has been lost. Early apoptotic cells are impermeable to the dye. The early stages of apoptosis are readily detectable using acridine orange.

### Determination of survival factors for colonic epithelial cells

The clonogenic adenoma cell line RG/C2 was used in a series of experiments to test for cytokines that could protect against apoptosis under conditions of serum withdrawal. The cells were seeded at a density of 10$^6$ cell per T25 flask. Three days after seeding, when the cells were in exponential growth, the medium was replaced after two washes with serum-free medium. Cells were fed with (1) standard culture medium (described above); (2) medium with all growth factors removed, i.e. DMEM without serum, insulin or hydrocortisone; (3) medium with all growth factors removed, but with the test agent added. For conditions (1) and (2) the appropriate solvent was added as a control. Test agents used were insulin (0.2 units ml$^{-1}$=7.692 μg ml$^{-1}$), the insulin-like growth factors IGF-I and IGF-II (50–200 μg ml$^{-1}$), hydrocortisone sodium succinate (1 μg ml$^{-1}$), epidermal growth factor (EGF) (10–40 ng ml$^{-1}$) and transforming growth factor β (TGF-β) (10 ng ml$^{-1}$).

### Western blotting

Samples were prepared from 10$^6$ whole cells and proteins were resolved by SDS–PAGE (10% acrylamide). c-*myc* was detected using the monoclonal antibody c-myc 3 (Oncogene Science, Cambridge, MA, USA).

**Figure 1** Bar charts showing apoptosis induced by deprivation of serum and growth factors in the adenoma cell line RG/C2 and protection conferred by IGF-I (100 μg ml$^{-1}$), IGF-II (100 μg ml$^{-1}$), insulin (0.2 units ml$^{-1}$), hydrocortisone (1 μg ml$^{-1}$) and EGF (10 ng ml$^{-1}$) but not by TGF-β (10 ng ml$^{-1}$). Apoptosis was measured as the percentage of cells shed into the medium. For each experiment, the cells were characterized for morphological characteristics of apoptosis by fluorescence microscopy of acridine orange-stained cells. This confirmed that the majority of the floating cells were apoptotic (80–90%) in both control and serum-deprived cultures. The results shown are means of at least three experiments. Experiments were conducted between passages 14 and 52. As after passage 52 the cells became resistant to apoptosis induced by serum withdrawal, a frozen stock was recovered and subsequent experiments were performed using these cells. This is the reason for the enhanced sensitivity to growth factor withdrawal seen in the EGF and TGF-β experiments

### RESULTS

### Cell–cell contacts as survival factors for colonic epithelial cells

Serial passage of the adenoma cell lines PC/AA, S/AN and PC/BH is possible using dispase which removes the cells as clumps, thereby retaining cell–cell contacts. When these cell lines are trypsinized and plated onto collagen they do not give rise to viable cultures (Paraskeva et al, 1984). We asked the question whether these cells with disrupted cell–cell contacts were undergoing apoptosis. To enable the cells to be readily collected, cells were maintained in

**Table 1** Experiments to determine if reduction of the serum concentration to 0.5% led to cell death

| Cell line | Time (days)[a] | Attached cell yield (×10$^6$) | | Percentage of cells floating | | *P*-value[b] |
|---|---|---|---|---|---|---|
| | | 20% FBS | 0.5% FBS | 20% FBS | 0.5% FBS | |
| RG/C2 | 2 | 4.05 ± 0.37 | 3.26 ± 0.38 | 11.2 ± 1.64 | 13.6 ± 1.95 | NS |
| | 3 | 5.24 ± 0.85 | 3.22 ± 0.59 | 10.8 ± 1.44 | 19.5 ± 2.15 | < 0.01 |
| | 4 | 5.12 ± 0.81 | 3.23 ± 0.23 | 17.3 ± 4.19 | 21.7 ± 2.70 | NS |
| AA/C1 | 2 | 3.22 ± 0.56 | 2.28 ± 0.45 | 3.45 ± 0.74 | 6.95 ± 0.09 | < 0.05 |
| | 3 | 3.82 ± 0.61 | 2.52 ± 0.51 | 4.33 ± 0.47 | 11.27 ± 1.37 | < 0.05 |
| | 4 | 5.00 ± 0.88 | 3.14 ± 0.84 | 2.62 ± 0.88 | 10.37 ± 1.96 | < 0.05 |
| PC/JW/FI | 2 | 4.24 ± 0.57 | 3.54 ± 0.43 | 8.25 ± 2.20 | 10.12 ± 1.07 | NS |
| | 3 | 5.06 ± 0.51 | 4.74 ± 0.54 | 8.51 ± 1.33 | 12.85 ± 2.22 | < 0.05 |
| | 4 | 4.91 ± 0.51 | 3.73 ± 0.69 | 12.21 ± 1.14 | 21.75 ± 2.60 | < 0.05 |

Two adenoma cell lines (RG/C2 and AA/C1) and one carcinoma cell line (PC/JW/FI) were used. The RG/C2 data are means ± s.e.m. of five experiments conducted between passages 37 and 45. The AA/C1 data are means ± s.e.m. of three experiments conducted at passages 82 and 83. The PC/JW/FI data are means ± s.e.m. of four experiments conducted between passages 86 and 88. The percentage of cells floating is used here as a measure of the extent of apoptosis in the culture. Apoptosis occurs spontaneously and after induction of apoptosis by reducing the serum concentration, shown by floating apoptotic cells which are shed into the medium (confirmed by the morphological analysis of the floating cells by acridine orange staining). [a] The time shown in days is the time since cells were placed in 0.5% serum and fresh medium was placed on the controls. [b] For the percentage of cells floating, levels of statistical significance were calculated using a two-tailed *t*-test for paired comparisons (NS, not significant).

 *© Cancer Research Campaign 1997*

**Figure 2** Fluorescence microscopy of RG/C2 (passage 19) attached and floating cells 24 h after serum and growth factor withdrawal. Cells were stained for 10 min with acridine orange in PBS (5 μg ml$^{-1}$). (**A**) Adherent control cells. (**B**) Adherent cells from serum-and growth factor-deprived cultures showing adherent apoptotic cells. (**C**) Floating apoptotic control cells. (**D**) Floating apoptotic cells from serum-and growth factor-deprived cultures. Bar = 100 μm suspension on dishes coated with polyHEMA, which prevents adherence. The cells are therefore also deprived of cell–matrix interactions. Under these conditions, an increase in apoptotic cells was detected 4 h after trypsinization for each of the cell lines (from 2.2% at time zero to 26.5% for PC/AA, from 2% at time zero to 15% for PC/BH and from 4% at time zero to 6.7% for S/AN). By 24 h, approximately 50% of the PC/AA cells (passages 22–23), 35% of the PC/BH cells (passages 61–64) and 40% of the S/AN cells (passage 65) were apoptotic.

The question remained as to whether the cells were undergoing apoptosis in response to disruption of cell–cell contacts or whether the apoptosis was due to lack of a suitable substrate, a phenomenon termed anoikis by Frisch and Francis (1994). The PC/AA line was selected for further study. Cells were dispased or trypsinized and plated onto either collagen- or polyHEMA-coated dishes. The dispased cells (i.e. those retaining cell–cell contacts) attached and grew on collagen as normal. Those dispased cells prevented from adhering by plating on polyHEMA remained largely viable, only 5–6% of the cells being apoptotic between 24 and 72 h, decreasing to 1.8% at 96 h as the cells started to grow in suspension. By 72 h, the trypsinized PC/AA cells plated onto polyHEMA were 72% apoptotic, compared with 0.58% at time zero. To determine whether cells permitted to adhere would be protected from apoptosis, cells trypsinized and plated onto collagen were examined. By 24 h, only 17% of the cells had attached, and the number of cells attached decreased to 11% at 72 h. Of the cells floating in the

**Figure 3** Reduction of c-myc levels after serum and growth factor starvation in the adenoma cell lines RG/C2 and BH/C1. Western blotting using the monoclonal antibody c-myc 3 (Oncogene Science) shows that c-myc is greatly reduced within 2 h of serum and growth factor deprivation. (**A**) RG/C2 passage 34. (**B**) BH/C1 passage 97. For both blots: lane 1, control (2 h); lane 2, serum-and growth factor-deprived cultures (2 h); lane 3, control (4 h); lane 4, Serum-and growth factor-deprived cultures (4 h); lane 5, control (6 h); lane 6, Serum-and growth factor-deprived cultures (6 h); lane 7, Control (24 h); lane 8, serum-and growth factor-deprived cultures (24 h)

medium, 85% were apoptotic by 72 h (using acridine orange–propidium iodide dual staining) and 97% were trypan blue positive. Therefore, by 72 h, most of the apoptotic cells were in a state of secondary necrosis, having permeable membranes. Seventy-two hours after seeding, the medium was therefore changed in remaining dishes, and the adherent cells were monitored for their fate. Nine days after seeding, the plates were examined. The cultures were composed of sparsely distributed colonies of flattened, apparently senescent colonies of around 16 cells in size. The results of this experiment suggest that, even if a collagen substrate is provided, most of the trypsinized cells die by apoptosis. Thus, it is the disruption of cell–cell contacts that leads to the death of these premaligant cells rather than the lack of a substrate. A small proportion of cells are rescued by the provision of collagen, but the evidence suggests that the majority of these cells will ultimately die by a process akin to senescence, and only a rare variant will give rise to a clonogenic cell line.

### Apoptosis in response to serum withdrawal

Preliminary experiments were carried out using three cell lines, the adenoma cell lines RG/C2 and AA/C1 and the carcinoma cell line PC/JW/FI, to determine if reduction of serum growth factors led to cell death and to find conditions that would enable us to look for specific survival factors. These experiments were conducted using 0.5% serum compared with the 20% serum used in the standard culture medium, and the attached and floating cells were counted separately. In colonic tumour cell cultures, cells spontaneously detach and float in the medium. Previously, we have shown these cells to be apoptotic (Hague et al, 1993), and there is evidence that these spontaneous apoptotic cells are the result of a terminal differentiation within the culture (Heerdt et al, 1994). Apoptosis induced by agents, such as the differentiation agent butyrate or γ-irradiation, increase the proportion of floating apoptotic cells within the culture (Hague et al, 1993; Bracey et al, 1995). In this study, therefore, apoptosis was measured as the percentage of cells floating in the medium, with confirmation that these cells were apoptotic by acridine orange staining and morphological examination for condensed chromatin.

Significant apoptosis above the spontaneous level was detected in all three of the cell lines after 3 days of treatment (Table 1), but for RG/C2 and PC/JW/FI the apoptosis induced at 2 days was not statistically significant (by two-tailed *t*-test for paired comparisons) and at the 4 day time point RG/C2 showed an increase in apoptosis, but not significant. It is noteworthy, however, that serum depletion had a greater effect on the attached cell yield of RG/C2 than on AA/C1 or PC/JW/FI. We hypothesized that 0.5% serum combined with the insulin and hydrocortisone supplements in the medium may be sufficient to partially protect the cells from apoptosis. We selected the RG/C2 adenoma cell line to determine if further apoptosis could be induced by removing the serum altogether and by omitting the insulin and hydrocortisone supplements.

### The identification of cytokines that can act as survival factors for colonic epithelial cells

Having shown that reducing the serum concentration to 0.5% leads to increased apoptosis in three cell lines, we went on to reduce the growth factor supplementation further by omitting the serum from the medium altogether and also by omitting the hydrocortisone and insulin. We chose one cell line, RG/C2, for further study. The complete absence of growth factor supplementation increased the extent of apoptosis obtained (compared with the experiments described above in which the serum was reduced to 0.5% and the insulin and hydrocortisone were present). This allowed the extent of apoptosis induced after 24 h of growth factor deprivation to be measured and the protective effects of individual growth factors to be assessed.

As explained previously, apoptosis was measured as the percentage of cells shed into the medium. The results of the experiments are shown in Figure 1. Under growth factor-deprived conditions, there was a significant increase in the proportion of cells floating in the medium. For each experiment the adherent and floating cells were characterized for morphological characteristics of apoptosis by fluorescence microscopy of acridine orange-stained cells. This confirmed that the majority of the floating cells were apoptotic (80–90%) in both control and serum-deprived cultures (Figure 2). The fact that the proportion of apoptotic cells in the floating cell population of the growth factor-starved cultures was not significantly different from the control cultures (i.e. from spontaneous levels of apoptosis) indicates that the cells shed as a result of treatment represent the induction of apoptosis. Figure 2D demonstrates the apoptotic nature of the floating cells induced by the treatment and Figure 2C shows the spontaneous floating apoptotic cells generated in the control culture. In the majority of experiments, in addition to an increase in the percentage of cells floating after serum and growth factor removal, apoptotic cells were also seen in the adherent cell population (Figure 2B). Taking the mean of all the experiments, the percentage of attached cells that were apoptotic increased from 1.54 ± 0.29% in the controls to 6.88 ± 1.33% in the serum and growth factor-deprived cultures.

The extent of apoptosis obtained was variable depending on the passage number of the cells. Experiments using insulin, IGF-I, IGF-II and hydrocortisone were conducted between passage 39 and passage 52. Experiments conducted to examine the effects of TGF-β and EGF were conducted with earlier passage cells (passages 16–17) that were more sensitive to growth factor withdrawal. These earlier passage cells were used because cultures at passage 53 were discarded as a result of the cells having reduced

© Cancer Research Campaign 1997

sensitivity to apoptosis induced by serum withdrawal. This emphasizes that cells can become independent of survival factors and that events in vitro may mimic tumour progression in vivo. It is notable that the RG/C2 cell line also became anchorage independent (able to grow in soft agar) at around the same passage number. As growth factors can often be limiting for growth in semi-solid medium (Peehl and Stanbridge, 1981), this may be because the cells have developed the ability to produce endogenous survival factors.

The growth factors, IGF-I and IGF-II, insulin, hydrocortisone and EGF were tested for their ability to protect cells from apoptosis under conditions of growth factor deprivation, along with the growth-inhibitory cytokine TGF-β, which does not induce apoptosis in RG/C2 (Hague et al, 1993). Insulin, IGF-I, IGF-II, hydrocortisone and EGF all acted as survival factors under these conditions, whereas TGF-β did not (Figure 1). Insulin was used at a concentration of 0.2 units $ml^{-1}$ (equivalent to 7.692 μg $ml^{-1}$), the concentration normally used in the medium to give improved colorectal epithelial cell cultures. Insulin was effective as a survival factor at this concentration. The bar charts (Figure 1) show the results of using IGF-I and IGF-II at 100 μg $ml,^{-1}$ in line with the concentrations found to be protective for oligodendrocytes (Barres et al, 1992). In both cases, this concentration provided protection from apoptosis on withdrawal of serum. Dose–response experiments showed that the 100 μg $ml^{-1}$ concentration was optimal for IGF-I (higher concentrations giving no further protection against apoptosis), however IGF-II provided further protection at 150 μg $ml^{-1}$ and, even more so, at 200 μg $ml^{-1}$, at which the proportion of cells floating was less than that obtained by culturing the cells in the standard complete medium (data not shown). It can be concluded that IGF-I is more potent than IGF-II, as IGF-I confers the greater protection against apoptosis at similar concentrations (Figure 1). (IGF-I and-II are of similar molecular weight and therefore the molarities used were similar.) Hydrocortisone and EGF both provided protection against apoptosis induced by serum withdrawal. Although EGF did not confer complete protection from apoptosis at 10 ng $ml^{-1}$ this concentration (for which the results are shown) was optimal for cell survival, 20 and 40 ng $ml^{-1}$ concentrations not being so effective. In our previous experiments, the growth-inhibitory cytokine TGF-β (10 ng $ml^{-1}$) did not induce apoptosis in the RG/C2 adenoma cell line in the presence of serum and growth factors (Hague et al, 1993). There remained the possibility, however, that TGF-β could protect against apoptosis by directing the cells into a quiescent state. However, Figure 1 shows that TGF-β did not protect against apoptosis under conditions of serum withdrawal. TGF-β is therefore a useful negative control in this assay. Each of the growth factors, with the exception of TGF-β, not only reduced the percentage of cells floating but also partially protected against the induction of apoptosis in the adherent population (data not shown).

Further to these experiments using the RG/C2 adenoma cell line, we have also repeated the insulin experiments using a newly isolated trypsinizable variant of PC/BH derived at passage 65 and designated BH/C1. This cell line also underwent apoptosis in response to growth factor deprivation (two- to sixfold increase), and insulin also acted as a survival factor in this cell line (data not shown).

### Western blotting showing down-regulation of c-*myc*

As deregulated c-*myc* can lead to apoptosis under conditions of serum deprivation (Evan et al, 1992), we examined whether serum and growth factor deprivation resulted in a downregulation of c-*myc* in the adenoma cell lines RG/C2 and BH/C1. The time points studied were 2, 4, 6 and 24 h after deprivation. Figure 3 shows reduced c-*myc* protein levels at all time points for both RG/C2 and BH/C1, including the 24-h time point used to assess the extent of apoptosis in the survival factor experiments previously described.

### DISCUSSION

Normal adult colonic epithelium is notoriously difficult to grow in culture. Limited success has been reported for short-term culture (2–4 days) (Buset et al, 1987) and, in our hands, using culture techniques developed for premalignant adenoma cells, normal colonic epithelial cells behave similarly (Paraskeva and Williams, 1992). We therefore hypothesize that specific survival factors must be required by normal colonic epithelium. The source of survival factors for colonocytes in vivo may be in the form of cytokines either in the systemic circulation or produced by neighbouring cells of different tissue type or in the form of cell–cell contact or cell–matrix contacts. Premalignant adenoma cells have, at least in part, overcome such restraints on cell survival. This is reflected in vitro by the fact that normal cells have a short lifespan, whereas adenoma cells have an extended lifespan and/or are immortal (Paraskeva et al, 1989*b*). However, the survival of adenoma cells in culture through maintenance of cell–cell contacts and their death following dissociation implies that some restraints on cell survival are still present particularly in the small premalignant adenomas.

Bedi et al (1995) have shown that normal colonic epithelial cells undergo apoptosis if disaggregated to single cells in a medium with low growth factor concentrations and that cells from tubular adenomas respond similarly. However, increasing survival was observed with progression through the adenoma to carcinoma sequence, tubulovillous adenoma cells having prolonged inhibition of apoptotic death after isolation and carcinomas having an even greater proportion of cells with the property of extended in vitro survival. Wang et al (1995) observed apoptosis in response to detachment of cells from the substrate using a calcium chelator. Our results using trypsin are similar. However, in these experiments, it was not possible to distinguish whether the substrate release or the disruption of cell–cell contacts was responsible for the induction of apoptosis (Wang et al, 1995). To address this difficulty, we prevented clumps of cells still retaining cell–cell contact from adhering by culturing them in polyHEMA-coated dishes. These cells remained viable as clumps and started to grow in suspension after an initial lag period, whereas the majority of trypsinized cells underwent apoptosis even when provided with a collagen substrate. These results suggest that dependence on substrate for survival is lost relatively early in colorectal carcinogenesis and that the premaligant adenoma cells are still largely dependent on cell–cell contacts for survival. In addition, Bates et al (1994) showed that dissociation of a colon carcinoma cell line, LIM 1863, which grew well in suspension, led to greater than 90% apoptosis after 12 h.

Bedi et al (1995) reported that the presence of APC mutations correlated with a decreased fraction of apoptotic cells at the time of biopsy and also after 8 h in culture. However, it is notable that cells carrying APC mutations still apoptose after dissociation to single cells. Adenomas from familial adenomatous polyposis (FAP) patients, although able to form cell lines if passaged as clumps of cells using dispase, still undergo apoptosis in response to trypsinization; for example, PC/AA and PC/BH were both derived

© Cancer Research Campaign 1997 

from patients with familial adenomatous polyposis (Paraskeva et al, 1984). APC mutations, therefore, do not confer resistance to cell death in response to loss of cell–cell contacts.

S/AN and PC/BH underwent apoptosis if cell–cell contacts were broken, however trypsinizable variant lines AN/C1 and BH/C1 were derived at passages 86 and 65 respectively. These cultures are able to grow after trypsinization to single cells, but this growth is dependent on a high density of cells. Even in the presence of 3T3 feeders, the cells are unable to grow if seeded at one million per T25 flask (results not shown). This may be because of the production of soluble growth factors by the epithelial cells which support their own survival.

Apoptosis is demonstrable on removal of the growth factors from the medium (fetal bovine serum containing undefined growth factors, hydrocortisone and insulin) in the premalignant cell lines RG/C2, AA/C1 and BH/C1 and in the carcinoma cell line PC/JW/FI, suggesting that exogenous survival factors are also important for cell viability. The adenoma cultures may therefore provide clues to the identity of some of the survival factors that are crucial to the survival of normal colonic epithelial cells. During colon carcinogenesis, some carcinomas clearly evolve mechanisms to evade apoptosis in response to growth factor depletion. For example, in our hands, HT29 does not undergo apoptosis on withdrawal of serum and growth factors in 24 h (results not shown). This may be because HT29 produces IGF-II and signals through the IGF-I receptor (Lahm et al, 1994). Such cell lines may therefore have evolved an autonomous survival pathway.

Given the potential importance of survival factors in the maintenance of tissue homeostasis and the previous reports of the potency of specific growth factors in protecting against apoptosis in other cell types, such as the oligodendrocytes (Barres et al, 1992), it is perhaps surprising that growth factors have not been tested for their ability to protect against programmed cell death in cells of colorectal origin, particularly with regard to the difficulty in growing normal colonic epithelium in vitro. To establish a system in which cytokines can be assessed for their effectiveness as survival factors, we chose the well-characterized adenoma cell line RG/C2 (Paraskeva et al, 1989*a*) and tested cytokines in serum- and growth factor-deprived conditions for their ability to suppress apoptosis. We chose to examine the IGF-I and -II for a number of reasons. IGF-I protects human erythroid colony-forming cells and human IL-3-dependent haemopoietic cells from apoptosis (Rodrigues Tarduchy et al, 1992) and both IFG-I and IGF-II are potent survival factors for oligodendrocytes (Barres et al, 1992). The presence of IGF-I and IGF-II receptors has been reported on normal adult human colonic epithelium (Rouyer-Fessard et al, 1990; Pillion et al, 1993) and both IGF-I and IGF-II are growth stimulatory for many human carcinomas, including colon (Lahm et al, 1994). These observations suggest that the IGFs may promote cell growth or survival in colonic epithelium under physiological conditions. Furthermore, it is thought that many colon carcinoma cell lines secrete biologically active IGF-I and -II producing a situation of growth autonomy. There is also the possibility that the production of IGFs by hepatocytes creates a suitable site for metastasis in the liver, as has been proposed for a lung carcinoma cell line that preferentially metastasizes to liver (Long et al, 1994), forming a paracrine regulation mechanism.

Both IGF-I and IGF-II protected against apoptosis induced by serum withdrawal, IGF-I being the more potent of the two. Insulin and hydrocortisone were tested for their ability to act as survival factors at the concentrations normally used for adenoma cell culture (see Methods), and at these concentrations both inhibited apoptosis. EGF protected against apoptosis but was less potent than the IGFs, insulin or hydrocortisone. This is in contrast to mammary epithelial cells in which EGF is a more potent survival factor than insulin (Merlo et al, 1995).

The observation that all of the growth factors tested (with the exception of TGF-β) can rescue the adenoma cells from apoptosis is of interest as the growth factors signal via very different receptors. The glucocorticoid hydrocortisone signals via nuclear receptors whereas the IGFs and EGF signal through surface receptors. The ceramide signalling pathway of apoptosis is triggered by agents that act at nuclear sites (e.g. 1,25 dihydroxyvitamin $D_3$, which acts on nuclear receptors, and γ-irradiation) as well as at cell-surface sites (e.g. TNF-α) (reviewed in Jarvis et al, 1996). It is therefore plausible that anti-apoptotic signalling may also be generated as a result of interaction at both cell surface and nuclear sites. It will be important to determine the mechanisms by which growth factors interrupt the apoptosis programme and whether such factors may interfere with anti-tumour therapies.

The question remains as to whether apoptosis in response to serum withdrawal would be a feature of normal epithelial cells or whether it is a consequence of the premalignant state. Askew et al (1992), working in a myeloid leukaemia cell system, and Evan et al (1992), working in Rat-1 fibroblasts, demonstrated that elevated c-*myc* levels induced apoptosis in serum-deprived cultures, whereas serum withdrawal in association with down-regulation of c-*myc* led to a quiescent state without apoptosis. This suggests that in these cell types apoptosis is not a natural response to growth factor deprivation but occurs as a result of inappropriate c-*myc* expression in the cells. Is this the case in colonic epithelial cells? Preston et al (1994) demonstrated that normal Syrian hamster cells do not die when serum is reduced to 0.2% but undergo growth arrest. In contrast, an immortalized variant at an early stage of preneoplastic progression underwent apoptosis in response to reduction of serum. Cells at a later stage of preneoplastic progression had a decreased susceptibility to apoptosis under these conditions. Our observations are that apoptosis is induced by serum deprivation in the premalignant adenoma cell lines AA/C1 and RG/C2 and also in the cell line from a carcinoma, PC/JW. However, some carcinoma cell lines were resistant to apoptosis in serum-free conditions in that we observed no induction of apoptosis in the cell lines HT29 or SW480 over a period of 24 h (data not shown). Harrington et al (1994), in addressing the question as to why serum deprivation induces apoptosis in Rat-1 fibroblasts that constitutively express c-*myc*, demonstrated that specific serum cytokines, i.e. insulin, IGF-I and -II and PDGF, protect cells from apoptosis after activation of the c-*myc* gene in serum-deprived conditions. Is the apoptosis in response to serum withdrawal a normal response or simply because of deregulated c-*myc* in our adenoma cell lines? To address this, we have shown that, after removal of serum and growth factors from cultures of RG/C2 and BH/C1, there is a rapid reduction in levels of c-*myc*. This is evidence that the c-*myc* gene is not deregulated in these cell lines and that the apoptosis detected upon serum and growth factor withdrawal is independent of c-*myc*. The fact that no further decrease in c-*myc* levels was seen between 2 h after serum withdrawal and 24 h after serum withdrawal suggests that the apoptosis obtained is not just the elimination of a subpopulation of cells with deregulated *myc*. It is therefore probable that normal colonocytes would respond in the same way as the adenoma-derived cultures and the survival factors demonstrated here may, therefore, play an important role in both normal and neoplastic colonocytes.

© Cancer Research Campaign 1997

In the case of the RG/C2 colonic adenoma cell line, the induction of apoptosis in response to serum and growth factor withdrawal does not involve wild type p53 as RG/C2 has only mutant p53 and no wild type (Baker et al, 1990). This finding is in agreement with observations in mammary epithelial cell lines (Merlo et al, 1995) which state that apoptosis in response to serum deprivation is independent of p53. The physiological response of colonic epithelial cells to growth factor deprivation probably involves a c-*myc*-independent and p53-independent pathway.

In summary, we previously observed that maintenance of cell–cell contacts is essential for the survival of early adenoma cells in culture (Paraskeva et al, 1984) and that the ability to survive after trypsinization to single cells represents an important stage in tumour progression (Williams et al, 1990). We have observed, in agreement with Bedi et al (1995), that disruption of early adenoma cells (i.e. those with low malignant potential) to single cells induces apoptosis. Retention of cell–cell contacts is therefore important for the survival of colonic epithelial cells. Late-stage adenoma and carcinoma cells that survive trypsinization have clearly evolved mechanisms to allow them to, at least in part, override the cell suicide response. These mechanisms remain to be elucidated. In contrast to early adenoma cells, normal colonocytes die by apoptosis soon after transfer into culture, even if cell–cell contacts are retained and despite the fact that the cells initially adhere and spread out. Premalignant adenoma cells are also induced to apoptose by the withdrawal of serum and growth factors. This apoptosis is not as the result of a conflicting signal in the form of high levels of c-*myc* and may represent the way in which normal cells would undergo apoptosis in the absence of the correct balance of survival signals. We have used this property of the cultures to examine growth factors for their ability to rescue the cells. Using the adenoma cell line RG/C2, we have found that IGF-I, IGF-II, insulin, hydrocortisone and EGF can act as survival factors for colonic epithelial cells, protecting the cells from apoptosis under conditions of growth factor withdrawal, whereas TGF-β does not protect under these conditions. With increasing passage number, the cells lose their requirement for survival factors. This may be another important event during colorectal cancer.

The ability to survive loss of cell–cell contacts and a reduced requirement for survival factors are markers of tumour progression (for example, late passage adenoma cells and some cancers). It will be important to determine the genetic changes that contribute to these phenotypes. Survival factor-dependent adenoma cell lines can be used to identify specific survival factors for the colonic epithelial cell. These studies may be important for elucidating the genes and survival factors that control cell survival in vivo and how defects in these control processes are involved in colorectal tumorigenesis.

### REFERENCES

Araki S, Simada Y, Kaji K and Hayashi H (1990) Role of protein kinase c in the inhibition by fibroblast growth factor of apoptosis in serum – depleted endothelial cells. *Biochem Biophys Res Comm* **172**: 1081–1085

Askew DS, Ihle JN and Cleveland JL (1993) Activation of apoptosis associated with enforced myc expression in myeloid progenitor cells is dominant to the suppression of apoptosis by interleukin-3 or erythropoietin. *Blood* **82**: 2079–2087

Baker SJ, Preisinger AC, Jessup JM, Paraskeva C, Markowitz S, Willson JKV, Hamilton S and Vogelstein B (1990) p53 gene mutations occur in combination with 17p allelic deletions as late events in colorectal tumorigenesis. *Cancer Res* **50**: 7717–7722

Barres BA, Hart IK, Coles HSR, Burne JF, Voyvodic JT, Richardson WD and Raff MC (1992) Cell death and control of cell survival in the oligodendrocyte lineage. *Cell* **70**: 31–46

Bates RC, Buret A, Van Helden DF, Horton MA and Burns GF (1994) Apoptosis induced by inhibition of intercellular contact. *J Cell Biol* **125**: 403–415

Bedi A, Pasricha PJ, Akhtar AJ, Barber JP, Bedi GC, Giardiello FM, Zehnbauer BA, Hamilton SR and Jones RJ (1995) Inhibition of apoptosis during development of colorectal cancer. *Cancer Res* **55**: 1811–1816

Berry RD and Paraskeva C (1988) Expression of carcinoembryonic antigen by adenoma and carcinoma derived epithelial cell lines: possible marker of tumour progression and modulation of expression by sodium butyrate. *Carcinogenesis* **9**: 447–450

Bracey TS, Miller JC, Preece A and Paraskeva C (1995) γ-radiation-induced apoptosis in human colorectal adenoma and carcinoma cell lines can occur in the absence of wild type p53. *Oncogene* **10**: 2391–2396

Brach MA, Devos S, Gruss HJ and Herrmann F (1992) Prolongation of survival of human polymorphonuclear neutrophils by granulocyte-macrophage colony-stimulating factor is caused by inhibition of programmed cell death. *Blood* **80**: 2920–2924

Buset M, Winawer S and Friedman E (1987) Defining conditions to promote the attachment of adult human colonic epithelial cells. *In Vitro* **23**: 403–412

Evan GI, Wyllie AH, Gilbert CS, Littlewood TD, Land H, Brooks M, Waters CM, Penn LZ and Hancock DC (1992) Induction of apoptosis in fibroblasts by c-myc protein. *Cell* **69**: 119–128

Frisch SM and Francis H (1994) Disruption of epithelial cell-matrix interactions induces apoptosis. *J Cell Biol* **124**: 619–626

Gavrieli Y, Sherman Y and Bensasson SA (1992) Identification of programmed cell death *in situ* via specific labeling of nuclear DNA fragmentation. *J Cell Biol* **119**: 493–501

Hague A, Manning AM, Hanlon KA, Huschtscha LI, Hart D and Paraskeva C (1993) Sodium butyrate induces apoptosis in human colonic tumour cell lines in a p53-independent pathway – implications for the possible role of dietary fibre in the prevention of large-bowel cancer. *Int J Cancer* **55**: 498–505

Hall PA, Coates PJ, Ansari B and Hopwood D (1994) Regulation of cell number in the mammalian gastrointestinal tract: the importance of apoptosis. *J Cell Science* **107**: 3569–3577

Harrington EA, Bennett MR, Fanidi A and Evan GI (1994) c-Myc-induced apoptosis in fibroblasts is inhibited by specific cytokines. *EMBO J* **13**: 3286–3295

Heerdt BG, Houston MA and Augenlicht LH (1994) Potentiation by short chain fatty acids of differentiation and apoptosis in human colonic carcinoma cell lines. *Cancer Res* **54**: 3288–3294

Jarvis WD, Grant S and Kolesnick RN (1996) Ceramide and the induction of apoptosis. *Clin Cancer Res* **2**: 1–6

Kyprianou N, English HF and Isaacs JT (1990) Programmed cell death during regression of PC-82 human prostate cancer following androgen ablation. *Cancer Res* **50**: 3748–3753

Kyprianou N, English HF, Davidson NF and Isaacs JT (1991) Programmed cell death during regression of the MCF-7 human breast cancer following estrogen ablation. *Cancer Res* **51**: 162–166

Lahm H, Amstad P, Wyniger J, Yilmaz A, Fischer JR, Schreyer M and Givel JC (1994) Blockade of the insulin-like growth-factor-I receptor inhibits growth of human colorectal cancer cells: evidence of a functional IGF-II-mediated autocrine loop. *Int J Cancer* **58**: 452–459

Long L, Nip J and Brodt P (1994) Paracrine growth stimulation by hepatocyte-derived insulin-like growth factor-1: a regulatory mechanism for carcinoma cells metastatic to the liver. *Cancer Res* **54**: 3732–3737

Merlo GR, Basolo F, Fiore L, Duboc L and Hynes NE (1995) p53-dependent and p53-independent activation of apoptosis in mammary epithelial cells reveals a survival function of EGF and insulin. *J Cell Biol* **128**: 1185–1196

Mesner PW, Winters TR and Green SH (1992) Nerve growth factor withdrawal-induced cell death in neuronal PC12 cells resembles that in sympathetic neurons. *J Cell Biol* **119**: 1669–1680

Muta K and Krantz SB (1993) Apoptosis of human erythroid colony-forming cells is decreased by stem cell factor and insulin-like growth factor-I as well as erythropoietin. *J Cell Physiol* **156**: 264–271

Paraskeva C and Williams AC (1992) The colon. In *Culture of Epithelial Cells*, Freshney RI (ed.), pp. 81–105. John Wiley & Sons: Chichester

Paraskeva C, Buckle BG, Sheer D and Wigley CB (1984) The isolation and characterization of colorectal epithelial cell lines at different stages in malignant transformation from familial polyposis coli patients. *Int J Cancer* **34**: 49–56

Paraskeva C, Finerty S, Mountford RA and Powell SC (1989*a*) Specific cytogenetic abnormalities in two new human colorectal adenoma-derived epithelial cell lines. *Cancer Res* **49**: 1282–1286

Paraskeva C, Harvey A, Finerty S and Powell SC (1989*b*) Possible involvement of chromosome 1 in *in vitro* immortalization: evidence from progression of a human adenoma-derived cell line *in vitro*. *Int J Cancer* **43**: 743–746

Peehl D and Stanbridge EJ (1981) Anchorage-independent growth of normal human fibroblasts. *Proc Natl Acad Sci* USA **78**: 3053–3057

Pillion DJ, Grizzle WE, Yang M, Meezan E, Stockard CR, Ganapathy V, Leibach FH, Myers RB and Haskell JF (1993) Expression of IGF-II/Man-6-P receptors on rat, rabbit, and human colon epithelial cells. *Am J Physiol* **264**: R1101–R1110

Preston GA, Lang JE, Maronpot RR and Barrett JC (1994) Regulation of apoptosis by low serum in cells of different stages of neoplastic progression: enhanced susceptibility after loss of a senescence gene and decreased susceptibility after loss of a tumor suppressor gene. *Cancer Res* **54**: 4214–4223

Raff MC (1992) Social controls on cell survival and death: an extreme view. *Nature* **356**: 397–400

Rodriguez-Tarduchy G, Collins MKL, Garcia I and Lopez-Rivas A (1992) Insulin-like growth factor-1 inhibits apoptosis in IL-3-dependent hemopoietic cells. *J Immunol* **149**: 535–540

Rouyer-Fessard CS, Gammeltoft S and Laburtha M (1990) Expression of two types of receptor for insulin-like growth factors in human colonic epithelium. *Gastroenterology* **98**: 703–707

Wagner AJ, Small MB and Hay N (1993) Myc-mediated apoptosis is blocked by ectopic expression of Bcl-2. *Mol Cell Biol* **13**: 2432–2440

Wang C, Eshleman JR, Willson JKV and Markowitz S (1995) Both transforming growth factor β and substrate release are inducers of apoptosis in a human colon adenoma cell line. *Cancer Res* **55**: 5101–5106Whitehead RH, Vaneeden P and Lukeis RE (1991) A cell line (LIM-2463) derived from a tubulovillous adenoma of the rectum. *Int J Cancer* **48**: 693–696

Williams AC, Harper SJ and Paraskeva C (1990) Neoplastic transformation of a human colonic epithelial cell line: *in vitro* evidence for the adenoma to carcinoma sequence. *Cancer Res* **50**: 4724–4730

Willson JKV, Bittner GN, Oberley TD, Meisner LF and Weese JL (1987) Cell culture of human colon adenomas and carcinomas. *Cancer Res* **47**: 2704–2713

Wyllie AH (1980) Glucocorticoid – induced thymocyte apoptosis is associated with endogenous endonuclease activation. *Nature* **284**: 555–556

© Cancer Research Campaign 1997


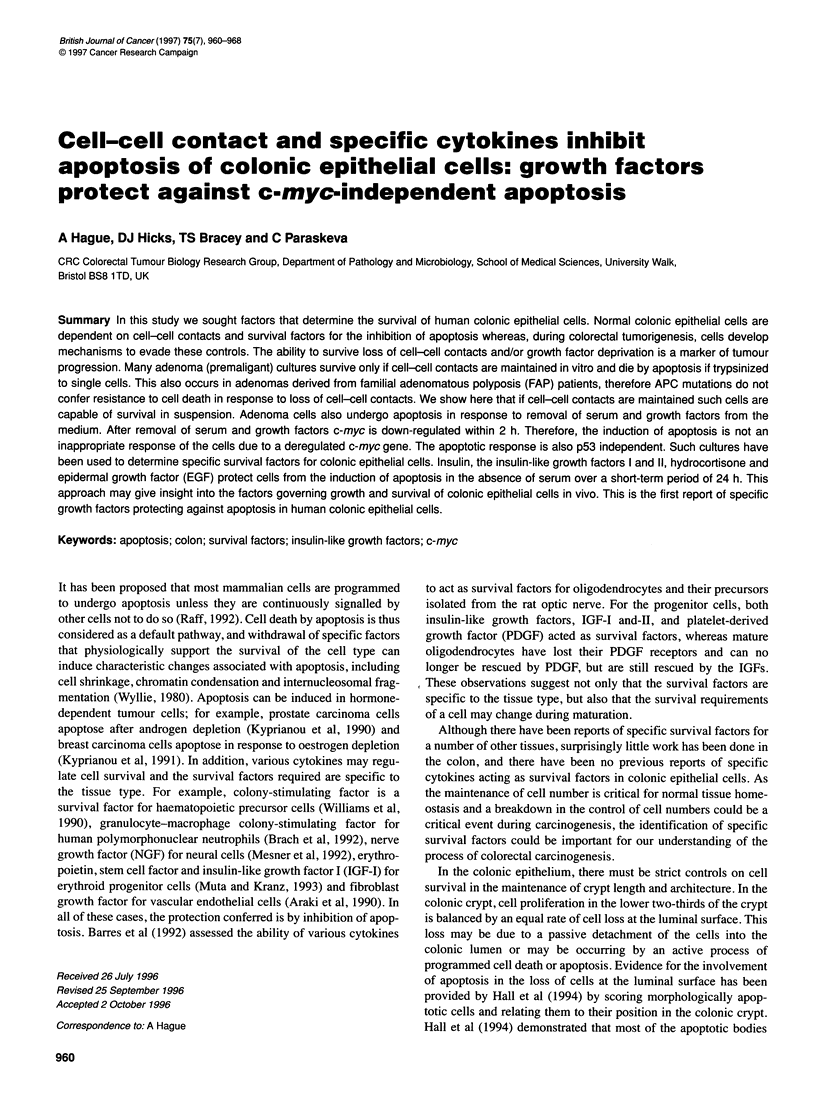

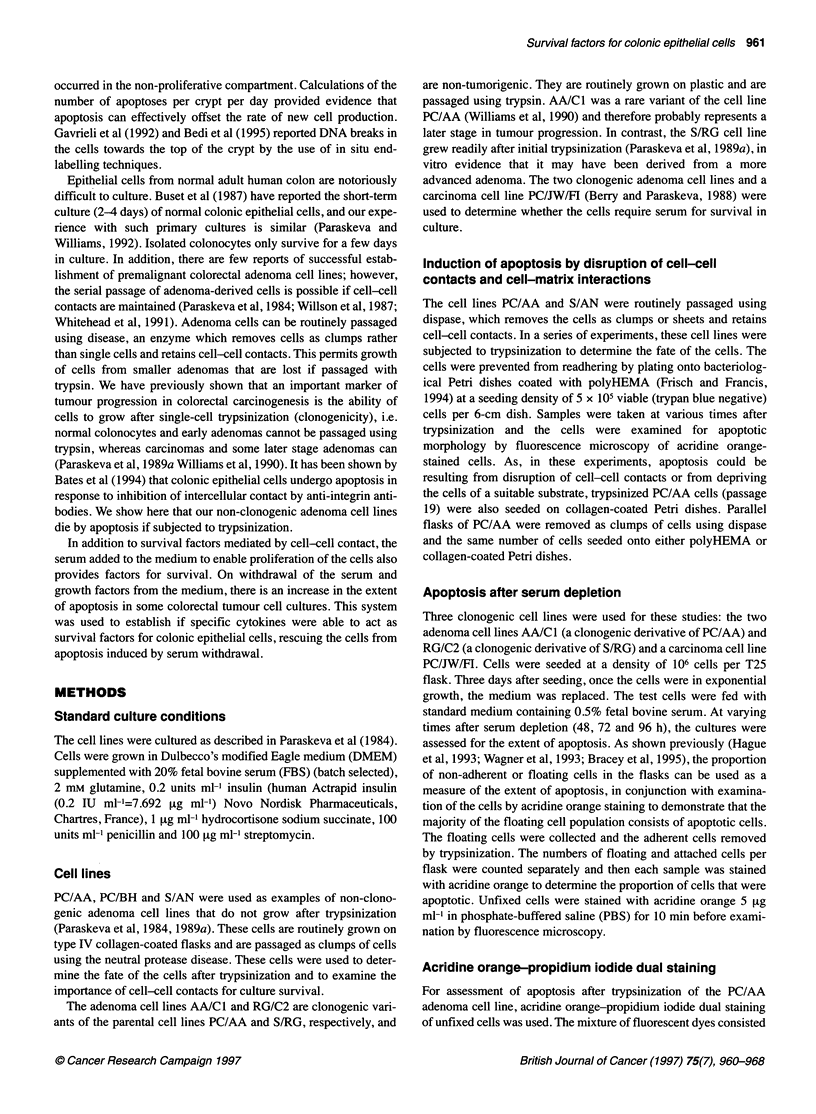

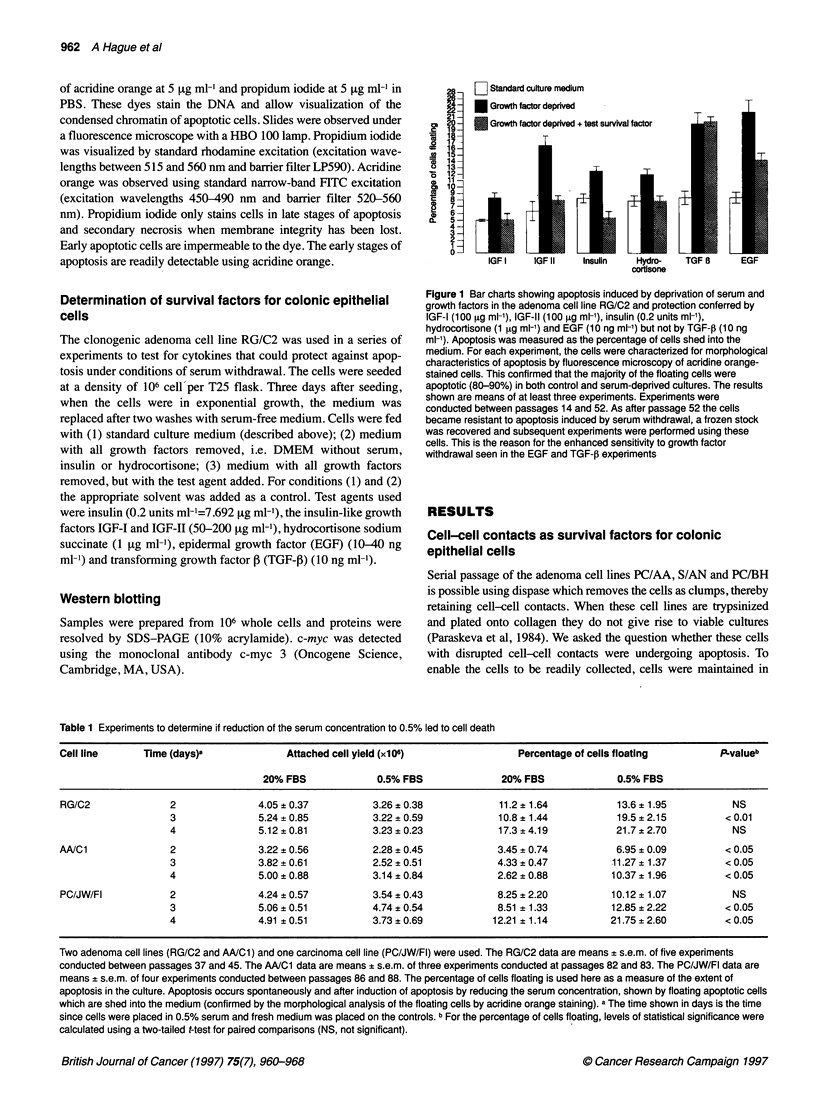

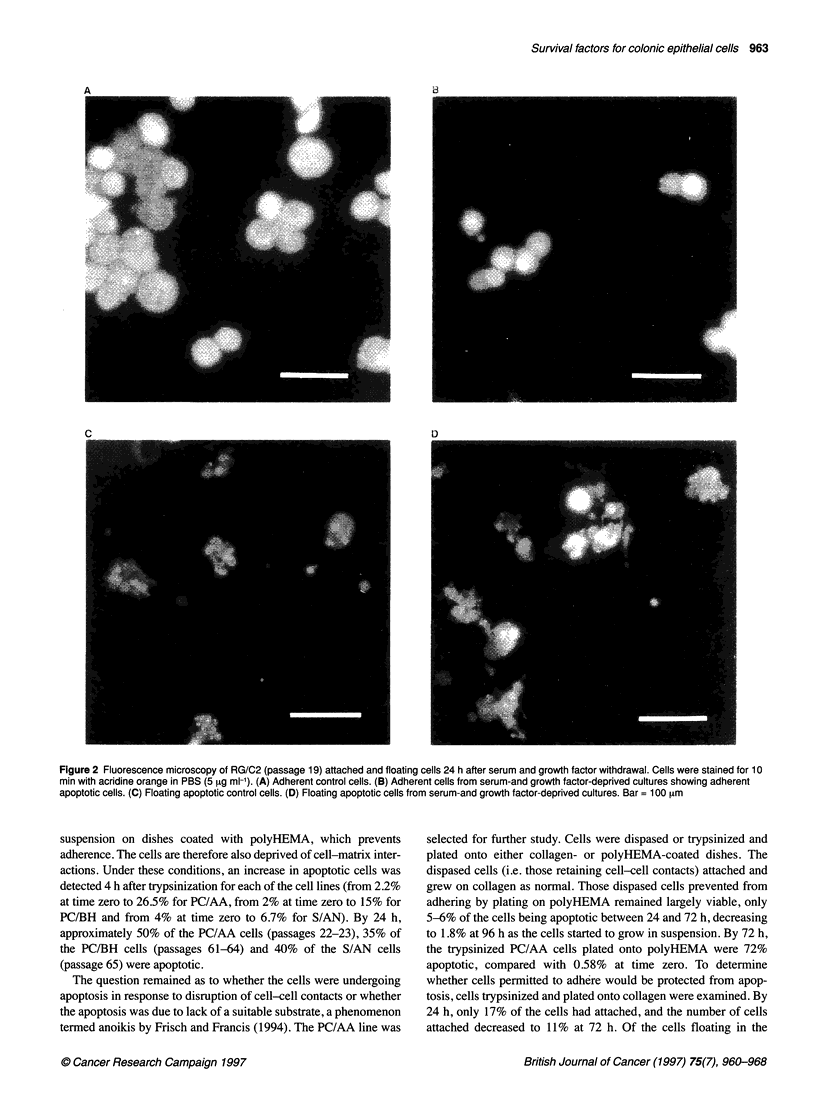

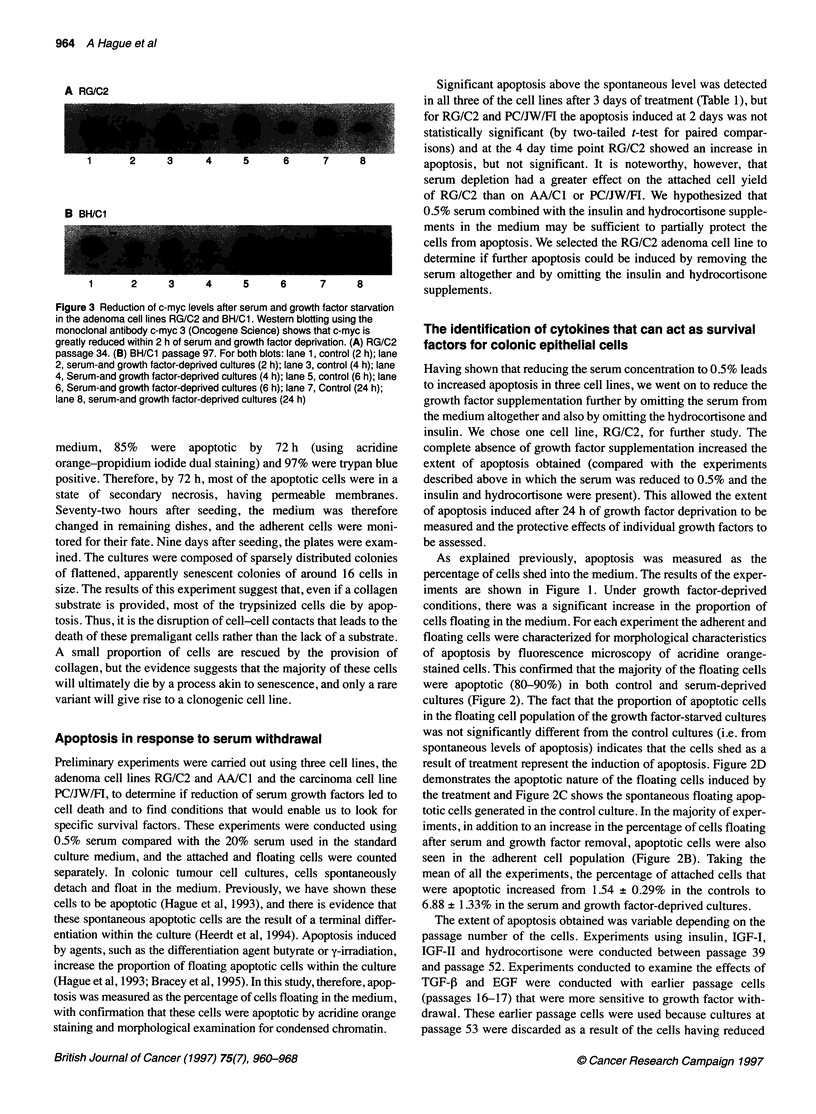

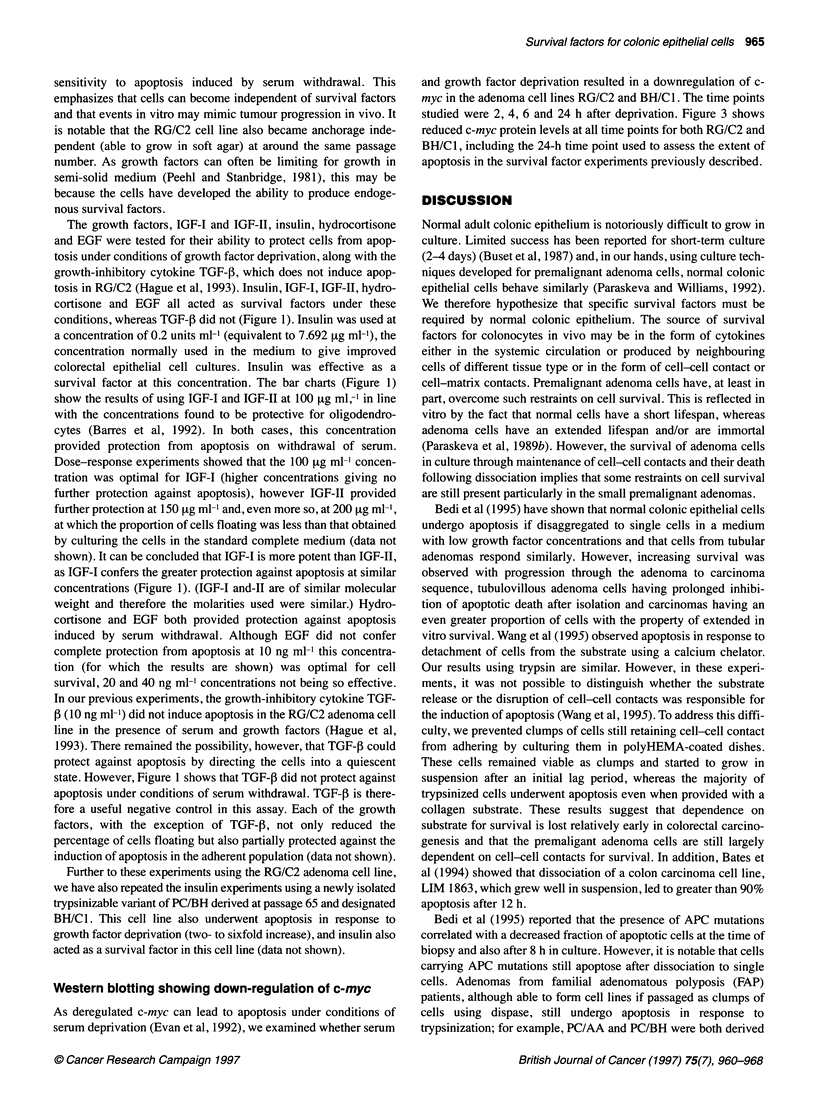

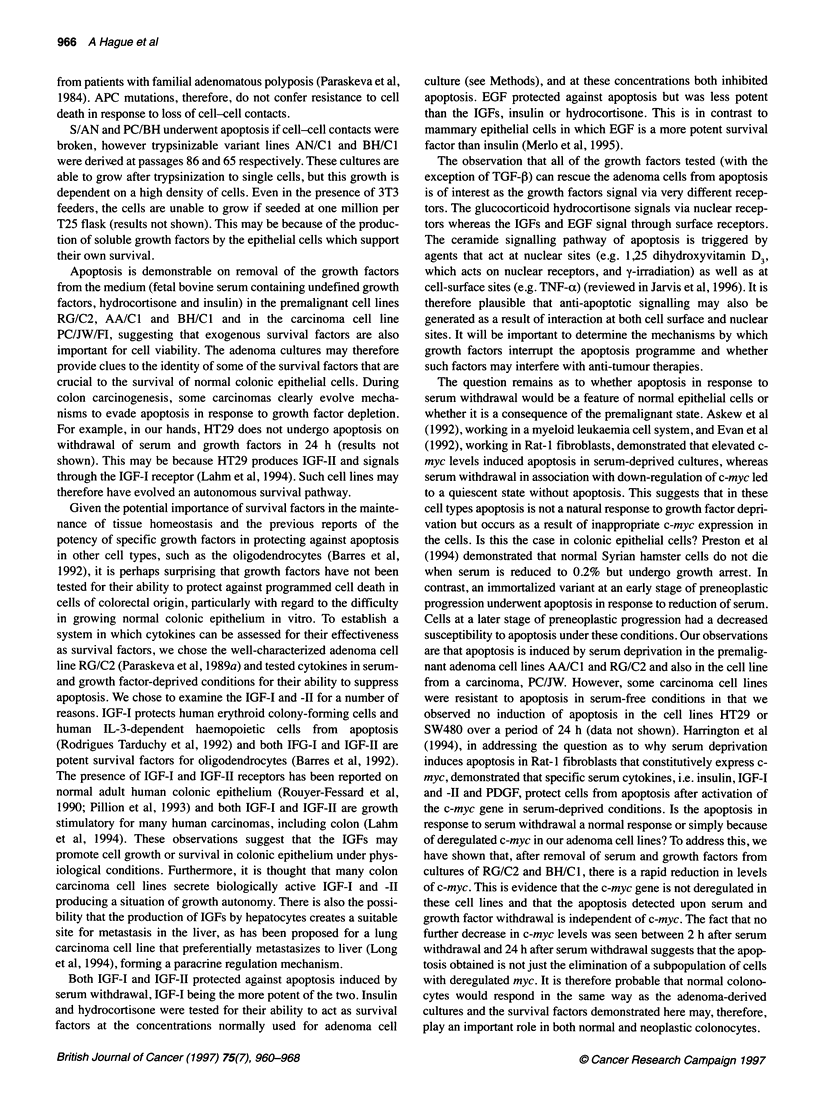

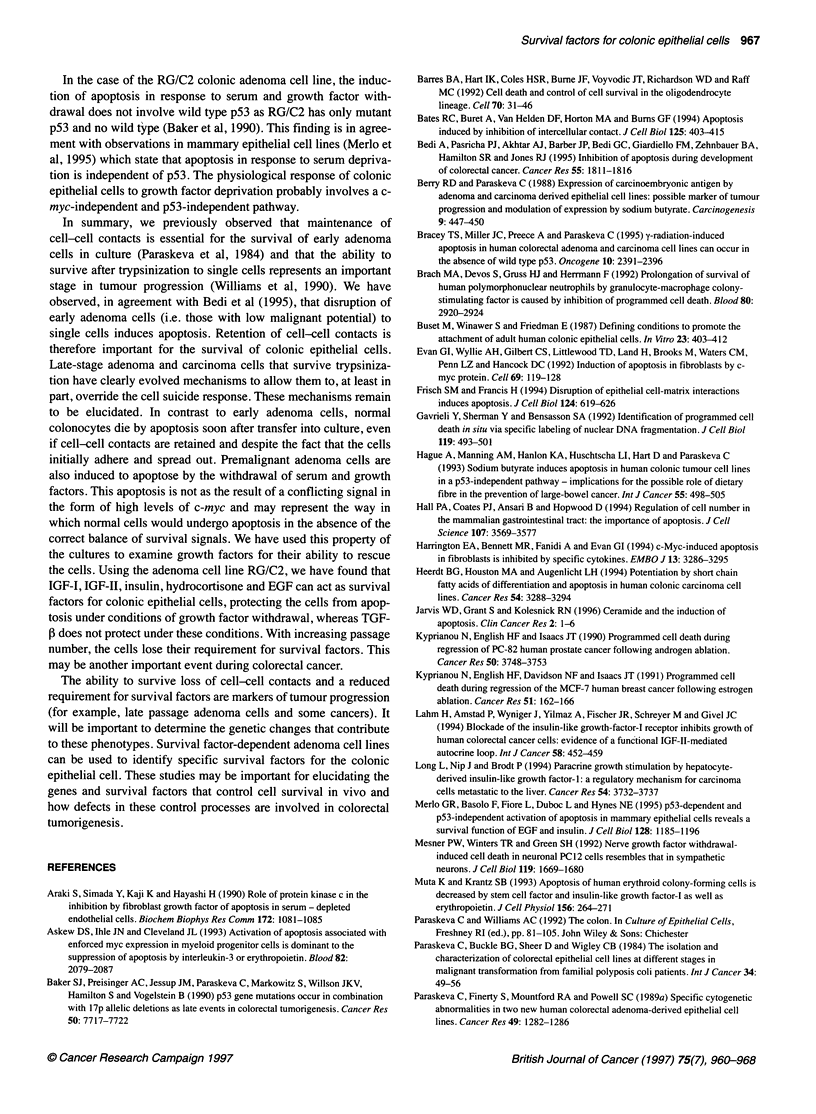

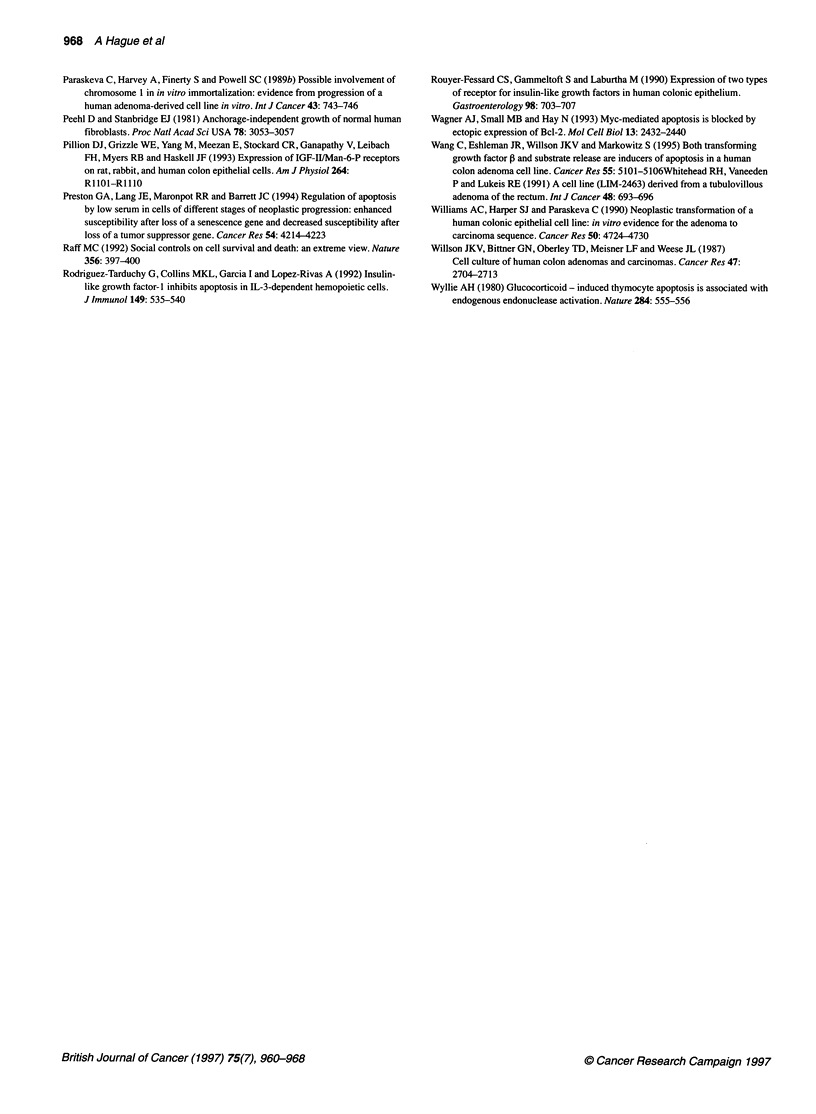


## References

[OCR_00871] Araki S., Simada Y., Kaji K., Hayashi H. (1990). Role of protein kinase C in the inhibition by fibroblast growth factor of apoptosis in serum-depleted endothelial cells.. Biochem Biophys Res Commun.

[OCR_00876] Askew D. S., Ihle J. N., Cleveland J. L. (1993). Activation of apoptosis associated with enforced myc expression in myeloid progenitor cells is dominant to the suppression of apoptosis by interleukin-3 or erythropoietin.. Blood.

[OCR_00882] Baker S. J., Preisinger A. C., Jessup J. M., Paraskeva C., Markowitz S., Willson J. K., Hamilton S., Vogelstein B. (1990). p53 gene mutations occur in combination with 17p allelic deletions as late events in colorectal tumorigenesis.. Cancer Res.

[OCR_00889] Barres B. A., Hart I. K., Coles H. S., Burne J. F., Voyvodic J. T., Richardson W. D., Raff M. C. (1992). Cell death and control of cell survival in the oligodendrocyte lineage.. Cell.

[OCR_00894] Bates R. C., Buret A., van Helden D. F., Horton M. A., Burns G. F. (1994). Apoptosis induced by inhibition of intercellular contact.. J Cell Biol.

[OCR_00898] Bedi A., Pasricha P. J., Akhtar A. J., Barber J. P., Bedi G. C., Giardiello F. M., Zehnbauer B. A., Hamilton S. R., Jones R. J. (1995). Inhibition of apoptosis during development of colorectal cancer.. Cancer Res.

[OCR_00903] Berry R. D., Paraskeva C. (1988). Expression of carcinoembryonic antigen by adenoma and carcinoma derived epithelial cell lines: possible marker of tumour progression and modulation of expression by sodium butyrate.. Carcinogenesis.

[OCR_00909] Bracey T. S., Miller J. C., Preece A., Paraskeva C. (1995). Gamma-radiation-induced apoptosis in human colorectal adenoma and carcinoma cell lines can occur in the absence of wild type p53.. Oncogene.

[OCR_00914] Brach M. A., deVos S., Gruss H. J., Herrmann F. (1992). Prolongation of survival of human polymorphonuclear neutrophils by granulocyte-macrophage colony-stimulating factor is caused by inhibition of programmed cell death.. Blood.

[OCR_00921] Buset M., Winawer S., Friedman E. (1987). Defining conditions to promote the attachment of adult human colonic epithelial cells.. In Vitro Cell Dev Biol.

[OCR_00925] Evan G. I., Wyllie A. H., Gilbert C. S., Littlewood T. D., Land H., Brooks M., Waters C. M., Penn L. Z., Hancock D. C. (1992). Induction of apoptosis in fibroblasts by c-myc protein.. Cell.

[OCR_00930] Frisch S. M., Francis H. (1994). Disruption of epithelial cell-matrix interactions induces apoptosis.. J Cell Biol.

[OCR_00934] Gavrieli Y., Sherman Y., Ben-Sasson S. A. (1992). Identification of programmed cell death in situ via specific labeling of nuclear DNA fragmentation.. J Cell Biol.

[OCR_00939] Hague A., Manning A. M., Hanlon K. A., Huschtscha L. I., Hart D., Paraskeva C. (1993). Sodium butyrate induces apoptosis in human colonic tumour cell lines in a p53-independent pathway: implications for the possible role of dietary fibre in the prevention of large-bowel cancer.. Int J Cancer.

[OCR_00945] Hall P. A., Coates P. J., Ansari B., Hopwood D. (1994). Regulation of cell number in the mammalian gastrointestinal tract: the importance of apoptosis.. J Cell Sci.

[OCR_00950] Harrington E. A., Bennett M. R., Fanidi A., Evan G. I. (1994). c-Myc-induced apoptosis in fibroblasts is inhibited by specific cytokines.. EMBO J.

[OCR_00954] Heerdt B. G., Houston M. A., Augenlicht L. H. (1994). Potentiation by specific short-chain fatty acids of differentiation and apoptosis in human colonic carcinoma cell lines.. Cancer Res.

[OCR_00959] Jarvis W. D., Grant S., Kolesnick R. N. (1996). Ceramide and the induction of apoptosis.. Clin Cancer Res.

[OCR_00968] Kyprianou N., English H. F., Davidson N. E., Isaacs J. T. (1991). Programmed cell death during regression of the MCF-7 human breast cancer following estrogen ablation.. Cancer Res.

[OCR_00963] Kyprianou N., English H. F., Isaacs J. T. (1990). Programmed cell death during regression of PC-82 human prostate cancer following androgen ablation.. Cancer Res.

[OCR_00973] Lahm H., Amstad P., Wyniger J., Yilmaz A., Fischer J. R., Schreyer M., Givel J. C. (1994). Blockade of the insulin-like growth-factor-I receptor inhibits growth of human colorectal cancer cells: evidence of a functional IGF-II-mediated autocrine loop.. Int J Cancer.

[OCR_00979] Long L., Nip J., Brodt P. (1994). Paracrine growth stimulation by hepatocyte-derived insulin-like growth factor-1: a regulatory mechanism for carcinoma cells metastatic to the liver.. Cancer Res.

[OCR_00984] Merlo G. R., Basolo F., Fiore L., Duboc L., Hynes N. E. (1995). p53-dependent and p53-independent activation of apoptosis in mammary epithelial cells reveals a survival function of EGF and insulin.. J Cell Biol.

[OCR_00989] Mesner P. W., Winters T. R., Green S. H. (1992). Nerve growth factor withdrawal-induced cell death in neuronal PC12 cells resembles that in sympathetic neurons.. J Cell Biol.

[OCR_00994] Muta K., Krantz S. B. (1993). Apoptosis of human erythroid colony-forming cells is decreased by stem cell factor and insulin-like growth factor I as well as erythropoietin.. J Cell Physiol.

[OCR_01003] Paraskeva C., Buckle B. G., Sheer D., Wigley C. B. (1984). The isolation and characterization of colorectal epithelial cell lines at different stages in malignant transformation from familial polyposis coli patients.. Int J Cancer.

[OCR_01010] Paraskeva C., Finerty S., Mountford R. A., Powell S. C. (1989). Specific cytogenetic abnormalities in two new human colorectal adenoma-derived epithelial cell lines.. Cancer Res.

[OCR_01019] Paraskeva C., Harvey A., Finerty S., Powell S. (1989). Possible involvement of chromosome 1 in in vitro immortalization: evidence from progression of a human adenoma-derived cell line in vitro.. Int J Cancer.

[OCR_01024] Peehl D. M., Stanbridge E. J. (1981). Anchorage-independent growth of normal human fibroblasts.. Proc Natl Acad Sci U S A.

[OCR_01028] Pillion D. J., Grizzle W. E., Yang M., Meezan E., Stockard C. R., Ganapathy V., Leibach F. H., Myers R. B., Haskell J. F. (1993). Expression of IGF-II/Man-6-P receptors on rat, rabbit, and human colon epithelial cells.. Am J Physiol.

[OCR_01034] Preston G. A., Lang J. E., Maronpot R. R., Barrett J. C. (1994). Regulation of apoptosis by low serum in cells of different stages of neoplastic progression: enhanced susceptibility after loss of a senescence gene and decreased susceptibility after loss of a tumor suppressor gene.. Cancer Res.

[OCR_01041] Raff M. C. (1992). Social controls on cell survival and cell death.. Nature.

[OCR_01045] Rodriguez-Tarduchy G., Collins M. K., García I., López-Rivas A. (1992). Insulin-like growth factor-I inhibits apoptosis in IL-3-dependent hemopoietic cells.. J Immunol.

[OCR_01050] Rouyer-Fessard C., Gammeltoft S., Laburthe M. (1990). Expression of two types of receptor for insulinlike growth factors in human colonic epithelium.. Gastroenterology.

[OCR_01055] Wagner A. J., Small M. B., Hay N. (1993). Myc-mediated apoptosis is blocked by ectopic expression of Bcl-2.. Mol Cell Biol.

[OCR_01059] Wang C. Y., Eshleman J. R., Willson J. K., Markowitz S. (1995). Both transforming growth factor-beta and substrate release are inducers of apoptosis in a human colon adenoma cell line.. Cancer Res.

[OCR_01064] Whitehead R. H., van Eeden P., Lukeis R. E. (1991). A cell line (LIM 2463) derived from a tubulovillous adenoma of the rectum.. Int J Cancer.

[OCR_01067] Williams A. C., Harper S. J., Paraskeva C. (1990). Neoplastic transformation of a human colonic epithelial cell line: in vitro evidence for the adenoma to carcinoma sequence.. Cancer Res.

[OCR_01072] Willson J. K., Bittner G. N., Oberley T. D., Meisner L. F., Weese J. L. (1987). Cell culture of human colon adenomas and carcinomas.. Cancer Res.

[OCR_01077] Wyllie A. H. (1980). Glucocorticoid-induced thymocyte apoptosis is associated with endogenous endonuclease activation.. Nature.

